# Improving the validity, relevance and feasibility of the continuum of care framework for maternal health in South Africa: a thematic analysis of experts’ perspectives

**DOI:** 10.1186/s12961-020-0537-8

**Published:** 2020-02-26

**Authors:** Mamothena Carol Mothupi, Lucia Knight, Hanani Tabana

**Affiliations:** University of the Western Cape, School of Public Health, Cape Town, South Africa

**Keywords:** Maternal health, continuum of care, framework, indicators, relevance, feasibility, validity, qualitative

## Abstract

**Background:**

The continuum of care is a key strategy for ensuring comprehensive service delivery for maternal health, while acknowledging the role of the social determinants of health. However, there is little research on the operationalisation of the framework by decision-makers and implementers to address maternal health challenges. The framework should be measurable and feasible for implementation in low- and middle-income country contexts. In this study, we explore experts’ perspective on monitoring indicators for continuum of care and key issues related to their use in the South African context.

**Methods:**

We conducted key informant interviews with a range of experts in decision-making and programme implementation roles in the health system and relevant sectors. Key informants provided their perspectives on systematically selected, nationally representative monitoring indicators in terms of validity, relevance and feasibility. We interviewed 13 key informants and conducted a thematic analysis of their responses using multi-stage coding techniques in Atlas.ti 8.4.

**Results:**

Experts believed that the continuum of care framework and monitoring indicators offer a multisectoral perspective for maternal health intervention missing in current programmes. To improve validity of monitoring indicators, experts suggested reflection on the use of proxy indicators and improvement of data to allow for equity analysis. In terms of relevance and feasibility, experts believe there was potential to foster co-accountability using continuum of care indicators. However, as experts stated, new indicators should be integrated that directly measure intersectoral collaboration for maternal health. In addition, experts recommended that the framework and indicators should evolve over time to reflect evolving policy priorities and public health challenges.

**Conclusion:**

Experts, as decision-makers and implementers, helped identify key issues in the application of the continuum of care framework and its indicators. The use of local indicators can bring the continuum of care framework from an under-utilised strategy to a useful tool for action and decision-making in maternal health. Our findings point to measurement issues and systematic changes needed to improve comprehensive monitoring of maternal health interventions in South Africa. Our methods can be applied to other low- and middle-income countries using the continuum of care framework and locally available indicators.

## Background

A review of national and global sources estimated that maternal mortality for South Africa was between 138 and 157.9 deaths per 100,000 live births in 2015 [1]. This is an improvement from the range of 140 to 174.1 deaths per 100,000 live births reported in 2013 [1]. The latest South African Demographic and Health Survey shows an increase in pregnancy-related mortality ratio (number of pregnancy-related deaths per 100,000 live births) from 150 in 1998 to 536 in 2016 [2]. Pregnancy related-deaths are classified as any deaths from any cause occurring during the ‘maternal risk period’ from pregnancy to 6 weeks postpartum [3]. The measure is sometimes used in South Africa due to challenges in estimation of the maternal mortality ratio [2]. Based on the resources the country invests in health, these estimates for maternal and pregnancy-related mortality should be lower [4]; they should reflect better progress towards the Sustainable Development Goals’ target for maternal mortality (a national goal of below 100 deaths per 100,000 live births) [5].

A continuum of care approach has been recommended for the country to improve maternal health outcomes [6–8]. Continuum of care for maternal health is the delivery of a myriad health promotion, preventive and curative interventions from pre-conception to postnatal care [7–9]. It is defined as “*access to care provided by families and communities, by outpatient and outreach services, and by clinical services throughout the lifecycle, including adolescence, pregnancy, childbirth, the postnatal period, and childhood. Saving lives depends on high coverage and quality of integrated service-delivery packages throughout the continuum, with functional linkages between levels of care in the health system and between service-delivery packages, so that the care provided at each time and place contributes to the effectiveness of all the linked packages*” ([9] p. 1359). Delivery of a continuum of care for maternal health is integrated with child health interventions, though in this study we focus only on the former. The aim of the continuum of care approach is to improve the efficiency and effectiveness of health service organisation; it increases the integration of services and reduces the duplication of effort, increases linkages between different stages and levels of care and, ultimately, improves health outcomes and outcomes of care [9, 10].

**Fig. 1:**
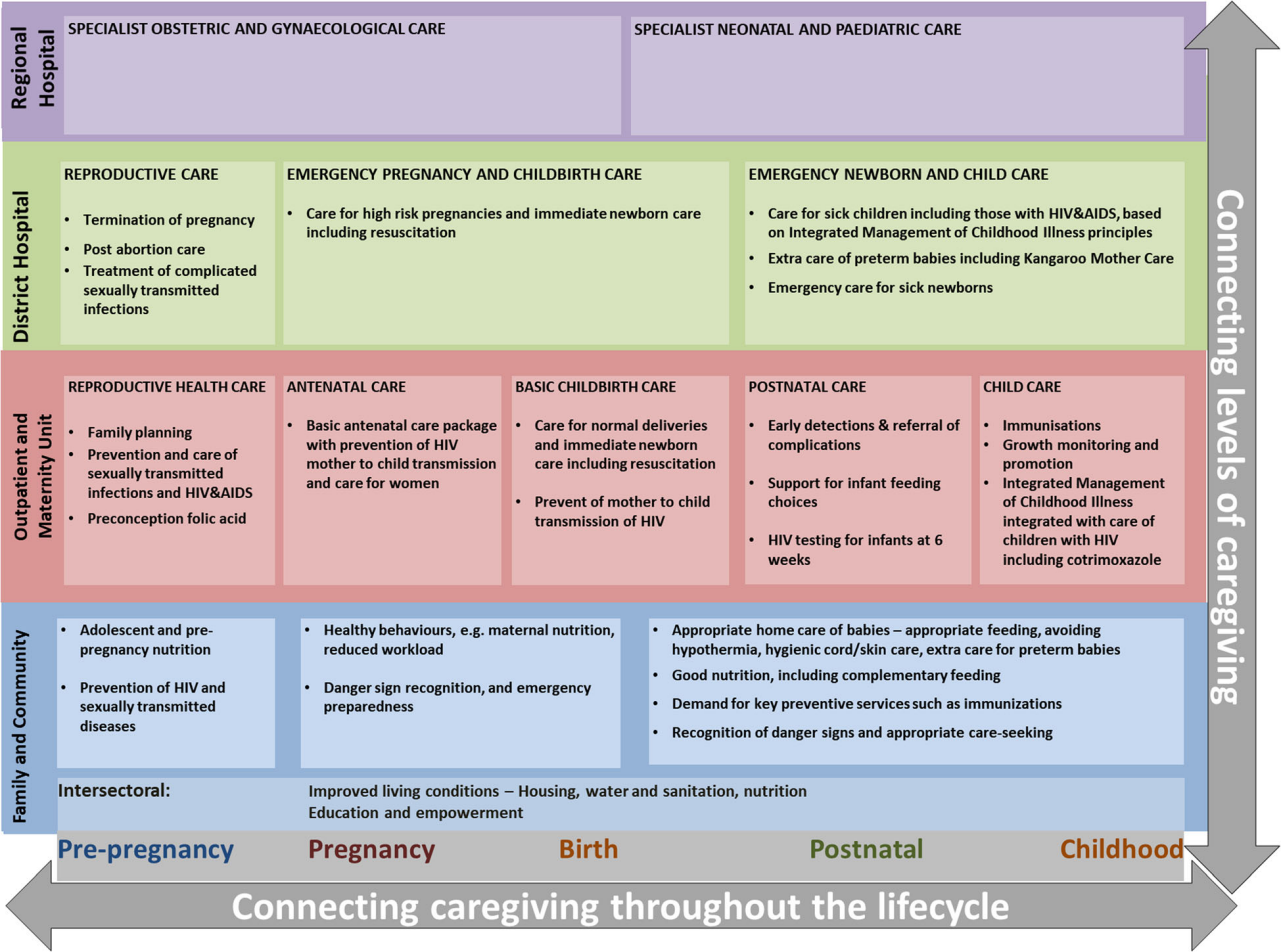
Continuum of care framework for maternal and child health in South Africa. The figure shows the packages of interventions necessary for provision to mothers over time and place. The vertical dimension is place, which ranges from family and community level to regional hospital. The horizontal dimension is time, which ranges from pre-pregnancy to childhood. Each place dimension is colour coded differently to highlight interventions needed at that level over time. Also crucial to the continuum of care are connecting the vertical levels of caregiving (place) and connecting caregiving over the lifecycle (time). The interventions outlined for each level come from evidence-based guidelines and were decided consultatively for the South African government by the relevant health, scientific and development bodies stated on the report. Fig. 1 is reproduced from a publicly available government report [7]. There are no permissions required to reproduce figures and tables produced by the National Department of Health and made available in public reports

National stakeholders in the South African health system (the Department of Health (DOH) and partner organisations) outlined a continuum of care framework as presented in Fig. 1 [7]. The framework serves to frame health policy and planning by promoting comprehensive service delivery involving all levels of the system across the lifecycle. It also highlights the importance of the social determinants of health acting at the community level of care to influence health outcomes. The social determinants of maternal health include structural determinants related to governance and policies as well as cultural and social values [11, 12]. In addition, the community context of living and other material conditions, social capital and social cohesion, health system, behavioural and psychosocial, and biological factors also influence maternal health outcomes as intermediary determinants [11, 12]. In the South African framework, there is a focus on ‘intersectoral factors’ that cross-cut a few social determinants of health. The South African health system aims to improve maternal health through multisectoral action to address the social determinants of health [6, 13, 14]. The current framework differs from other models that typically define the continuum of care for maternal health only from pregnancy to the postnatal period, with the exclusion social determinants [15].

The current strategic framework for maternal health in South Africa envisions future monitoring and evaluation of services from a continuum of care perspective [16]. Quality of care initiatives also recommend action on the continuum of care to improve maternal health in South Africa [6]. Although the strategic goal exists, as yet there is no defined approach for the planning of services and monitoring of performance along the continuum of care. There is a research gap in defining and validating a comprehensive set of indicators that can be used to assess and monitor the continuum of care for maternal health in the country. Implementing and monitoring the comprehensive set of services on the continuum will complement current quality of care efforts in the health system to improve maternal health outcomes.

In a preliminary study [17], we used the continuum of care framework (Fig. 1) to guide the identification of relevant indicators for all services, using local data sources. These data sources included the South African District Health Information System and household surveys. The aim was to propose a set of indicators for monitoring the continuum of care for maternal health in South Africa. The available indicators are currently used in programmes to monitor various maternal health and community interventions. In this study, we ask experts to reflect on their potential use within the continuum of care framework. Using existing indicators relieves the data collection burden from an already overwhelmed health system workforce.

For existing indicators to support policy and programme decision-making, they have to be valid, relevant and feasible in the new context for which they are intended [18–20]. To expand on the preliminary study that identified the indicators, in this study we aim to explore experts’ perspectives on key issues that arise in re-orienting indicators to monitor the continuum of care. The purpose of the study is to strengthen the continuum of care framework and improve its potential use in maternal health policy and planning. The study is part of a larger project to identify, evaluate and use available indicators to track continuum of care performance for maternal health in South Africa.

## Methods

### Aim

The aim of this study was to assess experts’ perspectives of the validity, relevance and feasibility of selected indicators and the indicator framework for the continuum of care for maternal health in South Africa. The study complements others in a larger project that identified the relevant indicators [15, 17] and will be used in the future to assess performance.

### Design

We conducted semi-structured key informant interviews with policy and programme decision-makers from the health system and other sectors. We focused on the framework and the domains of ‘empowerment’, ‘nutrition’ and ‘quality of care’. Our preliminary research had shown that ‘quality of care’ was a weak area of continuum of care measurement [15]; ‘empowerment’ and ‘nutrition’ are broad and multidimensional domains that needed further contextualisation for South Africa. The use of specific indicator domains also helps to delve into the thematic issues affecting the validity of the indicators in general. The individual indicators used present an opportunity for participants to elaborate by making concrete examples from the indicator framework.

### Preliminary research

Indicators were selected from local data sources using criteria developed by the authors. The criteria were (1) frequent data collection, (2) reliability, (3) already in use in policy- and decision-making in South Africa, (4) alignment with the continuum of care framework, and (5) nationally and sub-nationally representative data [17]. The National Indicator Data Set of the District Health Information System of South Africa was used to select health service indicators and the annual General Household Survey was used to select data on services related to social determinants of health [17]. These indicators are presented in Table 1.

**Table 1:** List of indicators across the continuum of care for maternal health in South Africa

	Continuum of care	Domain	Indicators	Definition	Source
1	Pre-pregnancy/Family and community	Reproductive health	Female condom distribution coverage	Number of female condoms distributed from a primary distribution site to health facilities or points in the community (e.g. campaigns, non-traditional outlets, etc.)	NIDS
2			Male condom distribution coverage	Number of male condoms distributed from a primary distribution site to health facilities or points in the community (e.g. campaigns, non-traditional outlets, etc.)	NIDS
3		Adolescent/Pre-conception nutrition	Mills fortification compliance rate	Operational flour and maize milling establishments that were compliant with fortification Regulation under the Foodstuffs, Cosmetics and Disinfectant Act (1972) as a proportion of milling establishments that were inspected	NIDS
4	Pre-pregnancy/Primary health facility	Reproductive health	Couple year protection rate	Women protected against pregnancy by using modern contraceptive methods, including sterilisations, as proportion of female population 15–49 years	NIDS
5	Pre-pregnancy/District hospital	Reproductive care	Rate of termination of pregnancy at 0–12 weeks	Pregnancies terminated in health facilities in the first 12 weeks of pregnancy as a proportion of total termination of pregnancies	NIDS
6			Rate of termination of pregnancy at 13–20 weeks	Pregnancies terminated in health facilities at 13–20 weeks of pregnancy as a proportion of total termination of pregnancies	NIDS
7			Rate of termination of pregnancy in women aged under 20 years	Termination of pregnancy in women aged under 20 years as a proportion of total termination of pregnancies in health facilities	NIDS
8	Pregnancy/Family and community	Healthy behaviours	OHH with pregnancy care rate	OHH with pregnancy care as proportion of households visited by the Ward-Based Outreach Team	NIDS
		Danger sign recognition and emergency preparedness			
9	Pregnancy/Primary health facility	Antenatal care (with PMTCT)	Rate of antenatal 1st visit before 20 weeks	Women who have a booking visit (first visit) before they are 20 weeks into their pregnancy as proportion of all antenatal 1st visits	NIDS
10			Antenatal 1st visit coverage	The proportion of potential antenatal clients coming for at least one (booking) antenatal visit; the census number of children under 1 year factorised by 1.15 is used as a proxy denominator – the extra 0.15 (15%) is a rough estimate to cater for late miscarriages (~10 to 26 weeks), stillbirths (after 26 weeks gestation) and infant mortality; pregnant women are regarded as potential antenatal clients from around 10 weeks’ gestation, i.e. spontaneous abortions before that as well as termination of pregnancy cases are excluded	NIDS
11			Antenatal client HIV re-test rate: retesting among positive HIV clients	Antenatal clients re-tested for HIV as proportion of antenatal clients tested negative for 1st HIV tests done during current pregnancy	NIDS
12			Percentage of antenatal clients starting on ART	Antenatal clients who started on ART as a proportion of the total number of antenatal clients who are HIV positive and not previously on ART	NIDS
13			Percentage of syphilis-positive pregnant women receiving Benz-penicillin 1st dose	Syphilis-positive pregnant women who received Benz-penicillin 1st dose as a proportion of pregnant women who tested positive for syphilis	DHIS
14			Percentage of syphilis-positive pregnant female receiving Benz-penicillin 2nd dose	Syphilis-positive pregnant women receive Benz-penicillin 2nd dose as a proportion of pregnant women who tested positive for syphilis	DHIS
15			Percentage of syphilis-positive pregnant women receiving Benz-penicillin 3rd dose	Syphilis-positive pregnant women receive Benz-penicillin 3rd dose as a proportion of pregnant women who tested positive for syphilis	DHIS
	Pregnancy/District hospital	Reproductive care	Emergency pregnancy care		
16	Birth/Primary health facility	Care for normal delivery and PMTCT	Delivery in facility rate	Deliveries in health facilities as proportion of expected deliveries in the population; expected deliveries are estimated as population under 1 year multiplied by 1.025 to compensate for stillbirths and infant mortality	NIDS
17	Birth/District hospital	Emergency childbirth care	Rate of caesarean section delivery	Delivery by caesarean section as a proportion of total deliveries in health facilities	NIDS
18	Post-natal care/Family and community	Recognition of danger signs and care-seeking	Rate of OHH with postnatal care	OHH with postnatal care provided to a mother and/or neonate within 6 days after delivery as proportion of households visited by the Ward-Based Outreach Team	NIDS
19	Post-natal care/Primary health facility	Early detection and referral of complications	Rate of mother postnatal visit within 6 days	Mothers who received postnatal care within 6 days after delivery as proportion of deliveries in health facilities	NIDS
20	Quality of care/Primary health facility	Cross-cutting	Rate for Ideal Clinic Status	Fixed primary healthcare facilities that have obtained Ideal Clinic status; Ideal Clinic status is a score of 70% or more on assessment of the facility readiness to provide good quality of care along the following main dimensions: 1. Administration, 2. Integrated Clinical Services Management, 3. Medicines, Supplies and Laboratory Services, 4. Human Resources for Health, 5. Support Services, 6. Infrastructure, 7. Health Information Management, 8. Communication, 9. District Health System Support, 10. Implementing Partners and Stakeholders	NIDS
21	Quality of care/District Hospital	Cross-cutting	Performance on National Core Standards	Hospitals that achieved a performance of 75% or more on National Core Standards self-assessment; National Core Standards measure: 1. Patient Rights, 2. Patient Safety, Clinical Governance and Clinical Care, 3. Clinical Support Services, 4. Public Health, 5. Leadership and Governance, 6. Operational Management, 7. Facilities and Infrastructure	NIDS
22	Linkages of care/Cross-cutting	Cross-cutting	Rate of obstetric clients transported	Obstetric clients as a proportion of total EMS clients transported	NIDS
23			Rate of rural obstetric response under 40 min	Primary obstetric calls responded to under 40 min in a rural area as a proportion of EMS P1 rural obstetric calls total	NIDS
24			Rate of urban obstetric response under 15 min	Primary Obstetric calls responded to under 15 min in an urban area as a proportion of EMS P1 urban obstetric calls total	NIDS
25			Rate of obstetric emergency rural inter-facility transfer under 60 min	Emergency obstetric inter-facility transfers response times under 60 min as a proportion of EMS obstetric rural inter-facility transfers	NIDS
26			Rate of obstetric emergency urban inter-facility transfer under 30 min	Emergency obstetric inter-facility transfers response times under 30 min as a proportion of EMS obstetric urban inter-facility transfers	NIDS
27	Cross-cutting/Community (intersectoral factors)	Water and sanitation	Environmental health: domestic water compliance rate	Domestic bacteriological and chemical water samples taken from Water Services Authorities and water service intermediaries at a point of use that conform to the standards set out in South African National Standard 241 for drinking water quality and safety as a proportion of water samples collected	NIDS
28		Water and sanitation	Percentage of women aged 15–49 years drinking safe water	Proportion of women aged 15–49 years in households that perceive their water to be safe	GHS
29			Percentage of women aged 15–49 years in households with adequate water infrastructure	Proportion of women aged 15–49 years in households with adequate water supply infrastructure	GHS
30			Percentage of women aged 15–49 years with basic sanitation facility	Proportion of women aged 15–49 years in households with basic sanitation facilities	GHS
31		Housing	Percentage of women aged 15–49 years with access to electricity	Proportion of women aged 15–49 years in households with access to electricity	GHS
32			Percentage of women aged 15–49 years living in adequate housing	Proportion of women aged 15–49 years living in households with ‘good’ or ‘very good’ wall, roof, and floor condition of the dwelling	GHS
33			Percentage of women aged 15–49 years living in formal housing	Proportion of women aged 15–49 years in housing classified as formal housing (by Regional Development Plan plan)	GHS
34		Nutrition	Percentage of women aged 15–49 years who have adequate food access	The mean proportion of women aged 15–49 years in households that ‘never’ had insufficient food, run out of money for food, cut the size of meals, skip a meal, or small variety of meals	GHS
35			Household Dietary Diversity Score	The Household Dietary Diversity Score by consumption of between 0 and 10 food groups, in households with women 15–49 years of age	GHS
36		Education	Percentage of women aged 15–49 years who are literate	Proportion of women aged 15–49 years who achieved grade 8 or more	GHS
37		Empowerment	Percentage of women aged 15–49 years with medical aid	Proportion of women aged 15–49 years who have medical aid	GHS
38			Percentage of women aged 15–49 years with income source	Proportion of women aged 15–49 years with at least one of social grant, working for wage/commission/salary, or involved in business activities	GHS

*ART* antiretroviral therapy, *DHIS* District Health Information System, *EMS* Emergency medical services, *GHS* General Household Survey, *NIDS* National Indicator Data Set, *OHH* Outreach to Households, *PMTCT* prevention of mother-to-child transmission

### Participants

We sought a purposeful sample of experts, with variation in experiences across maternal health policy and programmes, social determinants of health, monitoring and evaluation, and multisectoral collaboration. Our sample included five experts from the health system (national and provincial DOH) working in maternal health policy and programme design, monitoring and evaluation (M&E), and quality assurance; four academics with experience in maternal health and social determinants of health (such as public health nutrition and empowerment); and four experts with experience in multisectoral collaboration to address social determinants of health (from Department of Agriculture, Forestry and Fisheries (nutrition and food security division), bilateral and development organisations, and Department of Planning, Monitoring and Evaluation). All experts have experience working in South Africa or similar low- and middle-income country (LMIC) contexts.

### Data collection

We searched online sources, used the authors’ networks and snowballing techniques to identify experts (potential participants), and kept a list in Microsoft Excel 2010. Potential participants were contacted via email and those available were sent informational material ahead of the scheduled interview. The informational material introduced the study and provided the table of indicators on which experts based their responses (Table 1). The interview guide and consent form were also sent ahead of the interview. Before data collection, the interview guide was tested on four suitable experts that were not part of the study sample, and refined for clarity of questions. After refining the guide, the interviews were conducted among 13 experts between January and May 2019. We contacted 19 experts, 16 confirmed participation and only 13 were interviewed due to time constraints on experts’ part. One author (MM) interviewed the participants in face-to-face meetings and via telephone. The open-ended nature of interview questions as well as probing by the interviewer reduced telephone participants’ likelihood to put less effort in responses (satisficing). Interview questions were not of a personal or sensitive nature, thus reducing the social desirability bias and lack of trust that may occur in telephone interviews. Similarly, the nature of questions did not impede response quality due to the lack of anonymity in face-to-face interviews. The interviews lasted between 40 min and 1 hour. The researcher took notes during and immediately after interview using Microsoft Word 2010; data was entered into Atlas.ti 8.4 for coding and analysis.

### Data analysis

The three themes of validity, feasibility and relevance guided the design of the open ended interview guide and a priori coding of responses: these themes are defined in Box 1. Validity referred to experts’ opinion of the suitability of indicators to measure and monitor specific domains. Relevance and feasibility refer to experts’ perspective on whether the framework and its indicators can support action and decision-making in the health system as well as multisectoral collaboration for maternal health. These concepts were adapted from Blas et al.’s [18] study of the measurement and monitoring of social determinants of health in LMICs. In Box 1, we merge both technical (ease of data acquisition, analysis and interpretation) and policy and programmatic feasibility (messages from indicators are ‘communicable’ and ‘comprehensible’) as described by Blas et al. [18]. Other interpretations of validity may intertwine with relevance, and feasibility is an important consideration in the use of indicators [21].

We conducted in vivo coding of responses under the a priori themes, followed by closer analysis and open coding of emerging concepts. A third stage of coding involved the synthesis of emerging concepts into sub-themes (list coding), including any contrasting views. Multistage coding is an essential component of thematic analysis and helps with the layering of issues and development of themes in a transparent, rigorous manner [22]. One author was involved in the coding, and two authors were involved in the analysis.

### Trustworthiness

Although using experts’ perspectives to assess validity is considered the least rigorous validation method [23], it is the most justified in our study because the goal was to explore the framework and indicators by those likely to use them. In addition, all the indicators are already in use for decision-making in their relevant sectors. We used a transparent and systematic approach for selecting indicators. The approach used a systematic review to define the measurement model for the continuum of care for maternal health, local data sources for availability of indicators, and individual indicators based on evidence from published literature and policy documents [15, 17]. This study serves as a qualitative evaluative exercise meant to support the interpretation of the continuum of care framework and its indicators in the South African context.

### Reflexivity

The framing of questions for the interviews reflects authors’ knowledge and perspectives of what is important to explore given current policy directions for maternal health in South Africa and other LMICs. There are similarities between South Africa and other LMICs in terms of systemic priorities for addressing maternal health challenges such as action on social determinants of health as well as improved quality of care and monitoring and evaluation. The main areas of interest were defined in advance and thus influenced the emergence of sub-themes. We defined validity, reliability and feasibility in advance because indicators are quantitative measures whose quality can be assessed from specific perspectives. Without guiding themes, participants could have probably had broader interpretations of the indicators and indicator framework.

### Ethics

Institutional ethics clearance was obtained from the University of the Western Cape Biomedical Research Ethics Committee as well as the departments where experts’ worked (where prior permission was needed). All transcripts were confidentially kept and are accessible only to authors, and names of experts are not included in the analysis and reporting.

## Results

In this section, we discuss key cross-cutting issues raised by participants regarding the framework and indicators of the continuum of care for maternal health. These issues emerged as sub-themes under the overarching themes of validity, relevance and feasibility.

### Validity of the framework and indicators

According to participants, the framework and indicators provided a holistic, multisectoral perspective of interventions needed to improve maternal health in the country. Participants saw the framework as a good basis for thinking about future integrated monitoring and evaluation of maternal health. As a participant stated:

“*These frameworks have a role because multisectoral action plans need to be monitored … The frameworks are useful to groups that care about the holistic perspective.*” (Public health nutrition and M&E researcher, academic institution)

In multisectoral platforms, participants believe frameworks such as the continuum of care can help monitor interventions by different sectors towards improved maternal health outcomes. A participant with experience in multisectoral public health programmes elaborated:

“*Frameworks are useful for program design and to coalesce stakeholders around an idea; if there is buy-in they can get everyone aligned around the same goals and strategies … It has to be the foundational basis if it is to be useful for monitoring and evaluation.*” (Multisectoral programme specialist, development organisation)

Some of the indicators in the continuum of care framework already form part of national multisectoral strategic frameworks. From a multisectoral monitoring and evaluation perspective, a participant reflected:

“*Continuum of care would fall under the outcomes monitoring, especially related to the Medium Term Expenditure Frameworks (MTEF), which is what all departments use to set their strategic plans, including the Department of Health (DOH). So from the MTEF the DOH will get the target of reducing maternal mortality in order to contribute to the national development plan. But there are also indicators in outcomes monitoring that are monitored for the improvement of maternal health – and this is about ARV* [anti-retroviral drugs] *access, antenatal care, attendance of early postnatal care etc …*” (M&E specialist, Department of Planning, Monitoring and Evaluation)

The Medium Term Expenditure Framework is a multisectoral strategic budgeting framework used by the government to plan the country’s development through all its departments. Continuum of care, as a phenomenon, is seen as an outcome that can be monitored to track the collective action of sectors. This implies the need for formulation of a composite outcome indicator for the continuum of care from the existing set.

The validity discussion also led to the emergence of two sub-themes reflecting gaps and challenges perceived by participants, which were (1) the use of proxy indicators and (2) measurement of indicators for sub-groups within maternal health.

### Use of proxy indicators

Proxy indicators provide an indirect measurement of interventions and domains when direct data is not available. Participants felt that some of the proxy indicators used could not adequately measure interventions targeted for maternal health. An example was multidimensional quality of care or the ideal clinic status indicator (Indicator 20, Table 1). This indicator measures quality of care from different dimensions at the facility level. Although it captures some maternal health components, it more broadly indicates the quality environment in which women and other population groups receive care. Thus, the participants felt that the indicator should be supplemented by others that more specifically measured quality of care for maternal health. Similarly, the fortification of foods indicator (Indicator 3, Table 1) measures intervention at the community level, but not utilisation by women of reproductive age.

Participants felt that the use of proxy indicators represented a compromise when data is unavailable. In the context of the continuum of care, participants felt there needed to be a balance between collection of data on new, more direct indicators and their usefulness for decision-making. As a respondent stated:

“*These are good indicators and they are comprehensive enough because we don’t even want to come up with too many indicators. Dietary diversity score* [for example] *is based on food groups and although we may need to go deep into having macro- and micro-nutrient information, and measure the micro-nutrient adequacy score, these are used by specialists. What we have is adequate for programs and to inform us about the situation in South Africa.*” (Maternal health and nutrition researcher, academic institution).

Thus, there are indicators that may be useful for researchers but not policy and programme implementers. For example, the dietary diversity score (Indicator 35, Table 1) is used in research and interventions that improve food security for individuals and populations. In contrast, other indicators related to nutrition may be outside of the scope of monitoring inputs/interventions along the continuum of care for maternal health.

### Measurement for sub-groups

Participants reflected on measurement and monitoring of the indicators along the continuum of care, and the extent to which they represent services to sub-groups of women of reproductive age. As a non-homogenous population group for maternal health, women of reproductive age may have different barriers in accessing services along the continuum. For example, a participant states that the framework requires,

“… *tweaking of some indicators to reflect the context of measurement. For example, the accessibility of the facility to people with disabilities. In the context of maternal health language disability is the most critical than physical disability*” (Policy-maker, DOH).

Tweaking then implies modification or integration of indicators that reflect local public health needs and promotes equitable service provision. Another example stated by participants was services to younger mothers, whose indicators can measure the quality elements of youth friendliness and interpersonal communication between providers and patients (i.e. patient experience of care). When there is no sub-grouping, it may be difficult to design and monitor targeted services along the continuum of care that also address inequitable access. The rural and urban divide was another equity dimension mentioned by participants as influential on the social determinants of health and access to care, and thus important to assess from the continuum of care perspective. Some indicators, such as obstetric emergency transport in rural vs. urban areas (Indicators 22–26, Table 1), were a good demonstration of this stratification, according to participants. To the extent possible, other indicators should explore these sub-divisions.

In terms of equity and the social determinants of maternal health, a participant stated:

“*There is a balance between acknowledging social determinants of health are important and focusing on the interventions that the health system needs to do. For the most part, for example in maternal health, the health system works on their own evidence of what preventable causes of death are and addresses those. Social determinants of health are acknowledged. But we need an equity lens to the maternal mortality rates that can contribute to a reporting of the attribution of other determinants on outcomes.*” (Technical Advisor, DOH)

Thus, the health system can also integrate social determinants of health data to help attribute maternal health outcomes to interventions and exposures outside of the health sector.

In summary, the participants felt that the framework and its indicators were valid in terms of providing a broad multisectoral perspective of maternal health intervention necessary to achieve and attribute outcomes. Validity could be improved by disaggregation of indicators to reflect maternal health subgroups for equity analysis and collection of more direct data. However, the collection of more data should be an exercise in balancing indicator utility and their precision.

### Policy and programmatic relevance and feasibility

According to participants, the multisectoral nature of the indicators means that they can complement multisectoral collaboration for maternal health at strategic levels. Participants stated that there was various multisectoral work on-going between health and other sectors in South Africa such as agriculture (nutrition and food security) and social development (women’s empowerment). However, these were restricted to the operational level without shared monitoring and evaluation frameworks. According to participants, this leads to a fragmentation in goals and perpetuates the current ‘silo’ functioning of separate government departments. According to a participant:

“*From the health perspective it has been about getting other sectors to do what health wants them to do. Which might not be best approach and a stumbling block*” (Technical Advisor, DOH)

In contrast, a co-accountability mechanism for maternal health outcomes will use shared frameworks that integrate indicators from different sectors. According to participants, this solves the essential problem, whereby it is “… *difficult to get sectors to care about the work that other sectors do, especially their monitoring indicators*” (Public health nutrition researcher, academic institution). Using the example of the role of nutrition in maternal health outcomes, another participant stated that:

“*There are more than 60 programs in different government departments dealing with food security and nutrition, including health. Nothing much happens in multisectoral action – a coordinating department should be having an information management system as a resource for anyone who wants to do research on food security and health*” (Technical Advisor and Programme Co-ordinator, Department of Agriculture, Forestry and Fisheries)

According to the participant, this points to the lack of relevant shared platforms and tools for cross-sectoral learning and research for informed decision-making.

Two sub-themes emerged that relate to the main gaps and challenges related to relevance and feasibility of the framework and its indicators, which were (1) lack of indicators for intersectoral action and (2) the need for the framework to reflect the evolving policy and public health context in the country.

### Lack of indicators for intersectoral action

Participants reflected on whether the indicators adequately captured the interface between health and other sectors in multisectoral collaboration to improve maternal health outcomes. At the interface of the health system and other sectors, participants stated that the continuum of care framework should include indicators that measure intersectoral action. According to a participant:

“*This* [the framework] *doesn’t get at the intersectorality; thus, the multisectoral nature is clearly a second thought. There is no extra information on intersectoral expected interventions or domains …*” (M&E specialist, development organisation)

The inclusion of specific indicators for intersectorality, according to participants, may improve the relevance and use of the continuum of care framework in decision-making in the country. Intersectoral indicators can include indicators of co-coverage, which measure efforts of multiple sectors simultaneously. As a participant elaborates: “*A co-coverage indicator will, for instance, look at impact of agriculture, water and education, gender all together at the household level*” (Public health nutrition and M&E researcher, academic institution). This implies that a co-coverage indicator can be a composite metric of interventions by different sectors.

Besides intersectorality, participants also stated the need for harmonisation of definitions and metadata to improve cross-sectoral use and interpretation. As an example, a participant compared how different sectors may define ‘child’ – in civil registration as an individual below 16 years of age, in social development sector as below 18 years of age, and in the health sector the differentiation between children and adolescents. This logic can be applied to maternal health indicators within the continuum of care framework to ensure key concepts or sub-groups are described uniformly. Harmonisation and intersectorality can thus enhance the ability to use indicators within multisectoral collaboration for maternal health.

### The importance of local policy and public health context

According to participants, the relevance of the indicators can be enhanced by their alignment with current priorities for maternal health in the country. These priorities reflect subnational action and targets in maternal health services as well as the prevailing social determinants of health. In terms of services, participants noted gaps in measurement of maternal nutrition and mental health interventions at the health facility level. In terms of social determinants of health, participants reflected on the need for more empowerment factors and their impact on maternal health. In particular, issues of gender-based violence and coercion were relevant as they were prevalent in the country and affect women’s reproductive choices, pregnancy experiences, child health outcomes and maternal mental health. According to participants, the current framework should reflect these missing and crucial services and indicators. As one participant stated: “*It is important to have a framework, but the framework should not be set in stone and need to be updated every five years with latest data*” (Empowerment and maternal health researcher, academic institution). These data will be reflective of emerging policy issues and public health concerns, and thus keep the framework and its indicators relevant to context.

Another aspect related to new policy priorities is respectful maternal care, which emphasises positive birth experiences for women delivering in facilities. According to a participant, indicators are needed that monitor the continuum of care from the patient perspective – current indicators are heavily oriented towards provision of services, and not enough on how patients experience care. Participants raised more contextual issues such as health literacy challenges and women’s traditional roles in South African households, which is tied to their empowerment and may affect their health and nutrition outcomes. The reflection on relevance thus reveals entangled contextual factors and highlights their necessity if the framework is to continually reflect the environment it is intended to monitor. Some of these factors are already reflected in the framework (e.g. empowerment) and others need to be collected in future iterations as data becomes available (e.g. maternal mental health, respectful maternal care).

In summary, the relevance and feasibility of the framework is centred on the potential to flatten out the current vertical or ‘silo’ mentality of sectors, persistent even in multisectoral platforms. However, improvements need to be made to include indicators of concomitant impact of multiple sectors/intersectorality. Additionally, the framework should continue to evolve and reflect emerging policies, public health challenges and health services that impact maternal health outcomes in South Africa.

## Discussion

One of the important ways to improve maternal health outcomes in South Africa is to strengthen health service monitoring to support planning and accountability in the health system [24]. From a public health perspective, this also means acknowledging social determinants of health and their role in maternal health [25]. Our study assessed experts’ perspectives on potential monitoring indicators for the maternal health continuum of care in South Africa. Participants in our study believed that the continuum of care framework and the selected indicators provided a needed multisectoral perspective of maternal health interventions in South Africa. This multisectoral perspective, according to participants, can serve as a foundation for integrated monitoring of health and non-health sector interventions, and shared accountability for maternal health outcomes in the country.

The indicators appraised by participants in this study show that gaps remain in reporting services along the continuum of care. For the health sector, there are gaps in indicators because not all services are reported in the health system [26] and some are monitored through parallel information systems [27, 28]. An example is quality of care, whose detailed audits are reported elsewhere, and only composite indicators are included in the routine data set [29]. This affects accessibility of data [29] and the lack of indicators for the framework. Other gaps, as identified by participants, were a lack of indicators for experience of care, mental health and services to vulnerable groups (such as disabled women or young mothers). These gaps may be reflective of a lack of health service delivery in the country, not just reporting challenges. This is particularly true for maternal mental health services, which are lagging behind in primary healthcare in South Africa [30, 31].

The framework of indicators needs to be continually improved to include the missing indicators when services and data become available. However, participants also highlighted the importance of balancing the need for more data with the utility of the indicators from a decision-making perspective. The use of already available indicators for new monitoring and evaluation goals reduces the burden of data collection on health workers [32], and fulfils the goal of getting more intelligence out of the health data already available in South Africa [33]. Thus, we recommend the use of available data to measure and monitor the continuum of care, with a consultative process for additions/modifications to better align with evolving priorities.

The validity of indicators is affected by how well they measure the intended constructs. Sometimes, proxy indicators are used in the absence of direct measures. Participants believed that proxy indicators that reflect community coverage instead of the maternal health population should be removed. In the absence of alternatives, however, such proxy indicators can remain until future improvements in measurement are made. Validating maternal health indicators should be an on-going process of re-evaluation to ensure that indicators are truly reflective of constructs and context [21].

Besides validity and gaps in measurement, participants also reflected on the relevance and feasibility of available indicators. For relevance, participants stated that indicators should appropriately reflect current policy priorities, social issues and public health challenges. South Africa has unique problems in maternal health, including teenage pregnancy [34], gender inequality and gender-based violence [35], inequitable access and quality of maternal care as well as risky social exposures [36, 37]. There are age, race/ethnicity, residential, socioeconomic, disability and other differences in maternal health experiences among women in South Africa [36, 38–40]; indicators need to reflect these disparities in order to create buy-in from users and effectively support decision-making and action. To improve the relevance of available indicators and the framework as a whole, we recommend strengthening of the health information system to collect data on disaggregation variables and support equity analysis. The inadequacy of disaggregation data is a weakness of the current health information system in South Africa and other LMICs [1, 28, 41], and needs to be improved to support effective use of health service data in the future.

The feasibility of indicators was mainly tied to the perceived ease of use in intersectoral platforms, according to participants in this study. Additionally, there is a need to go beyond tracking sector performance individually and to integrate measurement of intersectoral collaboration for maternal health. Intersectoral collaboration refers to the mutually beneficial, integrated efforts of sectors who ideally share a common M&E framework [42]. According to our participants, intersectoral collaboration indicators would capture the true interface of the health system with other sectors. Intersectoral indicators are crucial because they have been used to address the social determinants of health and improve equity, accountability and planning for services in the era of the Sustainable Development Goals [18, 43–45]. Currently, intersectoral indicators for reproductive, maternal, and newborn, child and adolescent health are insufficiently measured and tracked [42]. Based on our findings, we recommend future research to document and develop indicators of intersectoral collaboration for inclusion in the continuum of care framework. In addition, we recommend future integration of data from other sectors and intersectoral collaboration into the health information system for accessibility and more comprehensive monitoring of interventions. The health system already acknowledges the need to integrate relevant non-health sector data and this needs to be implemented [13, 27].

The experts who participated in our study are stakeholders who have insight on the possible implementation challenges and measurement gaps in the indicator framework for the continuum of care. In this way, the study identifies key issues for improvement of the indicator framework to better support the goal for comprehensive monitoring and evaluation of maternal health in South Africa. We thus believe that this assessment of validity, relevance and feasibility of the indicator framework can be the basis for its future refinement and use in decision-making and action for maternal health in South Africa. Similar studies can be conducted in other LMICs to enhance monitoring and decision-making on multisectoral and comprehensive maternal service delivery. Studies should involve local stakeholders to help shape indicators that consider within-country priorities and support targeted decision-making.

### Limitations

Our study does not reflect all of the possible perspectives of stakeholders or experts in maternal health and determinants in South Africa. It is also not a consensus-building process on how to improve the continuum of care framework or on which final list of indicators to use. Rather, it is a reflection by diverse stakeholders to identify key issues that can make the indicator framework implementable. Our study is also not a final validation of the indicators, as it is part of on-going research that will also use quantitative methods to measure the available indicators and derive composite metrics to track progress in health system performance in South Africa.

## Conclusion

This study highlighted experts’ perspectives on the framework and indicators for the continuum of care for maternal health in South Africa. The study provided insights into the potential utility of the continuum of care framework to maternal health planning and programming in South Africa. It provides recommendations from the perspective of decision-makers and implementers to improve the use of valid, relevant and feasible indicators. The framework can be applied to assessment of comprehensive health services and monitoring and evaluation. Validity considerations focused on the adjustment of indicators to measure maternal health more accurately. Indicators were relevant from a multisectoral perspective but did not sufficiently reflect current policy directions and contextual factors. To improve feasibility, indicators should also be amenable to future monitoring of intersectoral collaboration to address the social determinants of maternal health. These findings help improve future measurement of indicators on the continuum of care for maternal health. They also point to systematic changes needed to improve monitoring of comprehensive maternal health service delivery in South Africa. These include future research in developing missing indicators, improved health service delivery, and strengthening health information systems to integrate data and indicators from other sectors. We recommend similar studies in other LMICs that involve stakeholders in repurposing and interpreting existing indicators, to reduce reporting burden and ultimately improve continuum of care monitoring in maternal health. These studies can support within-country improvements in service delivery across the continuum, which complement global, cross-national goals for maternal health.

Box 1 Definition of validity, feasibility and relevance themes that guided the first phase of thematic analysis (adapted from Blas et al. 2017 [18]) **Validity:** The suitability of indicators for measurement of their specific domain(s) and have a relationship with maternal health outcomes.

**Relevance:** The framework and indicators are seen as useful by decision-makers and implementers in the country and can support policy and programmatic action.

**Feasibility:** The information for the framework and indicators can be easily be understood, acquired and used for maternal health policy and programmes in the country.

## Supplementary information

**Additional file 1.** Interview guide.

## Data Availability

The datasets used and/or analysed during the current study are available from the corresponding author on reasonable request. No participant confidential information will be released.

## Abbreviations

DOH: Department of Health
LMICs: Low- and middle-income countries
M&E: Monitoring and evaluation
NIDS: National Indicator Data Set
