# Supplementary material for: Improving the validity, relevance and feasibility of the continuum of care framework for maternal health in South Africa: a thematic analysis of experts’ perspectives
Source: Health Res Policy Syst. 2020 Feb 26;18:28. doi: 10.1186/s12961-020-0537-8 (PMC7045428; doi:10.1186/s12961-020-0537-8)
Supplement: Supplementary file 1 — Additional file 1. Interview guide. [file 12961_2020_537_MOESM1_ESM.docx]

**INTERVIEW GUIDE**

Continuum of care is defined as:

*“…access to care provided by families and communities, by outpatient and outreach services, and by clinical services throughout the lifecycle, including adolescence, pregnancy, childbirth, the postnatal period, and childhood. Saving lives depends on high coverage and quality of integrated service-delivery packages throughout the continuum, with functional linkages between levels of care in the health system and between service-delivery packages, so that the care provided at each time and place contributes to the effectiveness of all the linked packages”^[[1]](#footnote-1)^*

The continuum of care is endorsed by the WHO and related bodies, as well as national governments including South Africa, as an important strategy to achieve maternal, neonatal and child health outcomes in particular. It is achieved by packages of interventions across the lifecycle and also includes the social determinants of health. Our research project aims to explore the measurement dimensions of the framework and identify useful, reliable indicators for the South African context. To do so we assessed health information system resources, particularly the DHIS and National Indicator Data Set for indicators relevant to maternal health services. We also assessed the annual General Household survey for indicators relevant to social determinants of health – or the intersectoral factors of housing, water and sanitation, empowerment, nutrition and education.

To gain deeper understanding of the concept of continuum of care for maternal health we conducted a systematic review^1^^[[2]](#footnote-2)^, focusing primarily on how it is defined and measured in low and middle income country contexts. Our synthesis of evidence revealed that there was a need for adequacy measurement on the continuum of care. Adequacy measures coverage of interventions, quality of care indicators (or proxies), incorporates social determinants of health indicators, and is also concerned about timeliness and linkages of care levels. In contrast, current measures used in research focus on coverage only, or effective coverage of limited set of interventions, no integration of SDH, and no exploration of the linkages of care indicators.

In the South African indicator framework we propose (attached in Appendix 1, with detailed descriptions of individual indicators in Appendix 2) [for manuscript, these are summarized as Table 1], based on currently available indicators from the DHIS and the GHS, we outline the set of indicators that can be used to monitor maternal health related performance by the health system and other sectors. In this study we focus on maternal health to demonstrate a case for measurement of a comprehensive set of indicators, and we recognize that maternal and child health should always be integrated. The set of indicators can ultimately be used to create a composite index that makes it easy to compare performance across districts/provinces.

The indicator framework (Appendix 1) can be related to other indicators of performance/ access to care in the health system, because it consists of already existing indicators. However it is also multisectoral in nature, in that it includes indicators related to social determinants of health. As a collective framework it repurposes current DHIS/NIDS indicators and GHS indicators for the purpose monitoring of maternal care access across a range of relevant sectors. However, it is a hypothetical framework. We need to think about the real world implications of it, as well as similar frameworks, for future multisectoral work to improve health outcomes.

**Question 1:** In the era of the SDGs and focus on wider determinants of health, what do you think is the role of frameworks that integrate health system and inputs from other sectors for monitoring healthcare and outcomes in South Africa, if any?

*For non-health sectors:*

**Question 1b**: In your perspective, what is your department’s role in collaboration with Department of Health to provide a continuum of care for maternal health in South Africa?

**Question 1c:** In what other ways is your department currently collaborating with health department if at all?

**Question 2a:** What do you think would be the strengths of using {Indicator X} for maternal health continuum of care domain {Y} specifically?

**Question 2b:** What do you think would be the weakness of using {Indicator X} for maternal health continuum of care domain {Y} specifically?

**Question 2c:** Which other domains do you think can be added to the framework?

**Question 2d:** Related to domains, which other indicators do you think are missing from the framework?

*Specific indicator(s) and domain(s) depend on expertise of the respondent.*

3.

The existing indicator gaps identified in the framework, as a result of lack of available data, affect the overall validity of the framework. This is particularly true for future use of indicators; if indicators are used to together formulate summary measures that can be used monitor health system performance and assess relationship with maternal health outcomes/trajectories.

**Question 3**: What practical steps would you recommend for the health system to address the existing indicator availability gaps as reported on the framework?

**4.**

Social determinants of health (SDH) are stated as one of the “critical success factors “ in the Strategic Plan for Maternal, Newborn, Child and Women’s Health and Nutrition. In our study we regard SDH indicators are an important part of a comprehensive framework for monitoring continuum of care for maternal health. The domains for SDH were set in earlier conceptual work by DOH and partners, although specific indicators were not specified/published. For the adequacy framework we sourced indicators from reliable national data sources, particularly the annual General Household Survey. These indicators imply the need for information from a diverse set of sectors, and the GHS has the benefit of being one central source of reliable information.

**Question 4a:** If social determinants of health are acknowledged as important, what in your view would constitute meaningful engagement of the health system with other sectors to address these?

**Question 4b**. What do you think the framework adds that the health system does not currently have in terms comprehensive monitoring indicators for maternal health?

**Question 4c**. What are the potential challenges of implementing frameworks that have both health and non-health sector indicators such as the current one?

**Question 4d.** To what extent can the framework in Appendix 1 be applied to intersectoral collaboration?

5. Do you have any questions/comments/concerns regarding the interview and/or the content we discussed

1. Kerber, K.J. et al., 2007. Continuum of care for maternal, newborn, and child health: from slogan to service delivery. *Lancet*, 370(9595), pp.1358–1369. [↑](#footnote-ref-1)
2. Mothupi, M., Knight, L. & Tabana, H., 2018. Measurement approaches in continuum of care for maternal health: a critical interpretive synthesis of evidence from LMICs and its implications for the South African context. *BMC Health Services Research*, 18(539), pp.1–9. [↑](#footnote-ref-2)
